# A quality-guided displacement tracking algorithm for ultrasonic elasticity imaging

**DOI:** 10.1016/j.media.2008.10.007

**Published:** 2009-04

**Authors:** Lujie Chen, Graham M. Treece, Joel E. Lindop, Andrew H. Gee, Richard W. Prager

**Affiliations:** Department of Engineering, University of Cambridge, Trumpington Street, Cambridge CB2 1PZ, UK

**Keywords:** Ultrasound, Elastography, Strain imaging, Tracking

## Abstract

Displacement estimation is a key step in the evaluation of tissue elasticity by quasistatic strain imaging. An efficient approach may incorporate a tracking strategy whereby each estimate is initially obtained from its neighbours’ displacements and then refined through a localized search. This increases the accuracy and reduces the computational expense compared with exhaustive search. However, simple tracking strategies fail when the target displacement map exhibits complex structure. For example, there may be discontinuities and regions of indeterminate displacement caused by decorrelation between the pre- and post-deformation radio frequency (RF) echo signals. This paper introduces a novel displacement tracking algorithm, with a search strategy guided by a data quality indicator. Comparisons with existing methods show that the proposed algorithm is more robust when the displacement distribution is challenging.

## 1 Introduction

After over 20 years of research and development, ultrasound elasticity imaging is gradually demonstrating its ability to characterize the mechanical properties of tissue. Research activity has progressed from simulation and laboratory experiments to clinical trials (Burnside et al., 2007). This trend is supported by ongoing development of signal processing methods and hardware innovation.

Some sort of deformation estimation is required by all elasticity imaging methods. For example, transient shear wave deformation induced externally (Sandrin et al., 2002; Bercoff et al., 2003) or by the acoustic radiation force (Sugimoto et al., 1990; Nightingale et al., 2001; Bercoff et al., 2004) can be used to obtain a two-dimensional map of the tissue’s mechanical properties. Depending on the magnitude of the deformation involved, some of these techniques may benefit from the displacement tracking algorithm that we describe in this paper. However, our focus is on the quasistatic approach to elasticity imaging (Ophir et al., 1991), where deformation is introduced by pressing on the probe. Two radio frequency (RF) ultrasound frames are acquired pre- and post-deformation. Some indication of the tissue elasticity is provided by the axial strain, which is usually retrieved in two steps^1^: *displacement estimation*, by matching pre-deformation RF data windows with post-deformation windows; and *strain estimation*, by differentiating the displacement field. The speed and accuracy of the strain imaging system depends heavily on the displacement estimation algorithm in the first step.

Among several techniques for displacement estimation (Langeland et al., 2003; Viola and Walker, 2003), correlation maximization was the first to be proposed (Ophir et al., 1991) and remains the most widely used. The pre-deformation RF frame is divided into an axial–lateral grid of small windows, which are associated with corresponding windows in the post-deformation frame by searching for the highest correlation match. Tissue displacement is then given by the shift between the pre- and post-deformation windows. Efforts have been made to adapt the correlation algorithm to provide subsample accuracy (Céspedes et al., 1995). Moreover, standard correlation, and its variants such as the sum of absolute differences and the sum of squared differences, have been integrated with multi-level or multi-scale schemes to improve the estimation stability (Chaturvedi et al., 1998; Yeung et al., 1998; Pellot-Barakat et al., 2004; Shi and Varghese, 2007). As an alternative to exhaustive search, Zhu and Hall (2002) proposed a tracking strategy that takes the displacement in each row of the grid as an initial guess for the next row. This allows a smaller search range and hence a reduction in the computational expense.

A second approach to displacement estimation is based on the phase of the RF data. O’Donnell et al. (1991, 1994) showed that the phase of the complex correlation between baseband signal windows is related to the relative displacement of the two windows. In particular, the correlation peak corresponds to zero phase, while a nonzero phase can be used to estimate the location of the peak without further search. Based on their work, Pesavento et al. (1999) proposed an improved method that calculates the displacement by tracking the zero phase location from point to point.^2^ The efficiency of this tracking approach lies in the way the displacement map is accumulated. A point’s displacement is initialized according to the value of a neighbour’s previously calculated displacement. Phase zero search at the new point then refines the accuracy of this initial guess. If the guess is close to the actual value, the correlation phase can be used to estimate the zero phase location with minimal further computation.

Although they employ different techniques for the point-wise displacement estimation, Zhu and Hall (2002) and Pesavento et al. (1999) both rely on a tracking strategy to advance the estimation process from one point to the next. This strategy sits on top of the low-level displacement estimation process, and controls the accumulation of the displacement map for the whole frame. As well as row-to-row tracking, there has been work on column-to-column (Jiang and Hall, 2007) and diagonal (Zahiri-Azar and Salcudean, 2006) strategies. Moreover, displacement tracking can be formulated as an optimization problem and solved by dynamic programming (Jiang and Hall, 2006; Rivaz et al., 2008). The main difficulty with all such schemes, however, is the implicit assumption that the displacement field is continuous. In practice, different layers of biological tissue have a tendency to slip over each other under mechanical load. Consequently, real displacement maps often exhibit several disjoint regions, with displacement continuity only within each region. Moreover, RF echo decorrelation is commonplace when scanning *in vivo*, producing regions of indeterminate displacement where continuity cannot be assumed. These factors present important challenges for displacement tracking algorithms.

When the continuity assumption is violated, a tracking algorithm might not only fail to find the correct displacement at any particular point, but also propagate this incorrect estimate into other parts of the image, producing so-called *drop-outs*. To guard against this, incorrect displacement estimates can be detected and replaced by values interpolated from nearby points, before they get a chance to propagate (Zhu and Hall, 2002; Jiang and Hall, 2007). Alternatively, a tracking algorithm might bypass poor estimates by allowing point initialisation within a larger, more flexible neighbourhood, as in the two-pass drop-out correction method of Lindop et al. (2008b), Treece et al. (2006, 2008). In the first tracking pass (from the top of the frame to the bottom), every point’s displacement is initialised from a seed point in the previous row. The algorithm examines a fixed-length lateral span in the previous row, centred on the current point, to locate the best seed point. In this context, ‘best’ is defined in terms of the correlation between the previously matched pre- and post-deformation RF windows. The second pass of tracking (from the bottom to the top) uses a similar seeding strategy and corrects remaining errors by comparing the correlations between the two passes: better correlated matches found in the second pass overwrite poorer matches discovered in the first pass.

One limiting factor of all current displacement tracking strategies is that the propagation direction, be it up–down, left–right or diagonal, is constrained. Some tracking errors might be avoidable by propagating the displacement estimates not from the predetermined fixed direction, where there might happen to be poor data, but from some other direction, where the data might be of better quality. In this paper, we develop this hypothesis into a novel, quality-guided displacement tracking algorithm for ultrasonic strain imaging. Its basis is a propagation scheme that is not constrained to any particular set of directions, but is instead guided by the quality of the data. The algorithm can be seeded at a single location or, for greater robustness, at multiple points. The quality measure that guides the algorithm can be any suitable metric calculated from the RF data. The paper includes a series of experiments that demonstrate the new method’s robustness in comparison with alternative approaches. Specifically, the quality-guided algorithm is able to track through geometrically irregular and disjoint regions, and copes well with regions of poorly correlated RF data.

## 2 Quality-guided displacement tracking

### 2.1 Seed initialization

The proposed quality-guided tracking algorithm comes in single-seed and multiple-seed variants. The former demonstrates the fundamental structure of the tracking strategy, while the latter is a significant extension that adds robustness when there are decorrelated regions and displacement discontinuities. For both versions, the first stage is seed initialization.

The seed initialization method used in this study is based on a simple grid test. On an RF frame, a grid of *N* regularly spaced points are tested as seed candidates. At each test point, a window is defined with the point at its centre. The magnitude of the complex cross-correlation is used to measure the similarity between the corresponding windows in RF frames recorded before and after deformation:

(1) $$|C(d_{x}\text{,}d_{y})|=\left|\frac{\underset{x\text{,}y\in W}{\sum }a_{1}(x\text{,}y)a_{2}^{*}(x+d_{x}\text{,}y+d_{y})}{\sqrt{\underset{x\text{,}y\in W}{\sum }|a_{1}(x\text{,}y){|}^{2}\underset{x\text{,}y\in W}{\sum }|a_{2}(x+d_{x}\text{,}y+d_{y}){|}^{2}}}\right|$$

where *C*(*d*_*x*_, *d*_*y*_) is the correlation coefficient at axial displacement *d*_*y*_ and lateral displacement *d*_*x*_, *a*_1_ and *a*_2_ are analytic RF signals obtained before and after deformation respectively, $a_{2}^{*}$ is the complex conjugate of *a*_2_, and *W* is the window size. A brute force search that varies (*d*_*x*_, *d*_*y*_) within the maximum expected displacement identifies the best match. The corresponding correlation coefficient magnitude and displacement are recorded for each test point. The search has a precision of one sample axially and one line laterally. After all grid points have been processed, the one with the highest correlation is chosen as the seed for the single-seed tracking algorithm. For the multiple-seed tracking algorithm, all of the grid points are used as seeds.

It remains to discuss the choice of an appropriate value for *N*, which is the algorithms’ only user-defined parameter. For the single-seed algorithm, the larger the value of *N*, the greater the chance of discovering a good seed. For the multiple-seed algorithm, *N* can be thought of as a natural scale parameter. With small *N*, the algorithm can seed and subsequently recover the displacement in a small number of large, disjoint regions. Should the user require greater sensitivity to smaller, disjoint regions, *N* should be increased. In the experiments reported in Section 3, we set *N* to 20, at which level the algorithm is capable of seeding regions occupying around 5% of the frame (the precise number depends on the regions’ shape, so this is just a rule of thumb). At this order of magnitude, we shall see that seeding has little impact on the overall computational load, which is dominated by the subsequent displacement tracking stage.

### 2.2 Single-seed, quality-guided tracking

The proposed tracking strategy requires a quality indicator to determine each point’s reliability before it is used to initialize a neighbouring point. A number of criteria may be used as quality indicators, including phase gradient variance and correlation coefficient. In this paper, all studies make use of the complex correlation coefficient between aligned pre- and post-deformation RF windows, as given in Eq. (1). The quality map may be represented as *Q*(*x*, *y*), where *y* and *x* are indices in the axial and lateral directions respectively. The tracking algorithm maintains a set *S* which contains points that have been initialized and are ready for processing. At the start of the first step, *S*_1_ (1 is the index of the step) contains the one seed, *p*_1_ = (*x*_0_, *y*_0_). As *p*_1_ is the only point ready for processing, it is selected and fed into any suitable displacement estimation method (Ophir et al., 1991; O’Donnell et al., 1991, 1994; Pesavento et al., 1999; Langeland et al., 2003; Viola and Walker, 2003) to calculate the displacement at this point. If using a correlation-based quality indicator, the calculated displacement (*d*_*x*_, *d*_*y*_) is then used to determine *Q*(*x*_0_, *y*_0_) according to Eq. (1).

In the second step, the point set is updated by removing *p*_1_ from *S* and adding its four neighbours, whose initial displacement estimates and qualities are assigned according to *p*_1_’s values:

(2) $$S_{2}=\{p_{2}\text{,}p_{3}\text{,}p_{4}\text{,}p_{5}\}$$

where *p*_2_ = (*x*_0_ + 1, *y*_0_), *p*_3_ = (*x*_0_ − 1, *y*_0_), *p*_4_ = (*x*_0_, *y*_0_ + 1), and *p*_5_ = (*x*_0_, *y*_0_ − 1). Since there are now four points ready for processing, the next point to be processed is selected according to the maximum quality criterion:

(3) $${\mathrm{Pt}}_{2}=\arg\max(Q(p_{2})\text{,}Q(p_{3})\text{,}Q(p_{4})\text{,}Q(p_{5}))$$

where *Pt*_2_ refers to the current point to be processed (2 denotes the index of the step), and the arg max( ) operator extracts the point with the maximum quality value from *S*. If there is a tie, as there will inevitably be at step 2, any one of the tied points is selected at random. Subsequently, *Pt*_2_ is processed by a displacement estimation method, and during this procedure both its displacement and quality are refined.

Similarly, in the third step, *Pt*_2_ is removed from the point set *S*. Its neighbours that have not yet been encountered are initialized with *Pt*_2_’s displacement and quality, and added to *S*. A new current point is then selected, according to the maximum quality criterion. This recursive process continues and can be described as

(4) $$S_{k+1}=S_{k}+\text{Neighbour}({\mathrm{Pt}}_{k})-{\mathrm{Pt}}_{k}$$

(5) $${\mathrm{Pt}}_{k+1}=\arg\max(Q(p)|p\in S_{k+1})$$

where the Neighbour( ) operator extracts a point’s 4-way neighbours that are not in *S* and have not yet been processed. For any neighbour that is already in *S*, a comparison is made between its quality value and that of the current point. If the current point’s quality is greater, the neighbour’s initial displacement estimate is replaced by the current point’s displacement. This simply reflects the fact that a better displacement estimate is now available. The algorithm terminates when the point set *S* is empty, which indicates that all points in the RF frame have been processed.

**Fig. 1:**
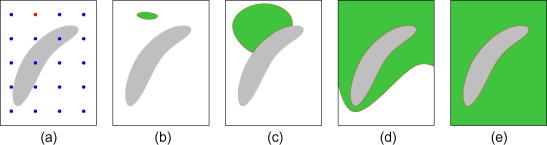
Illustration of the single-seed, quality-guided tracking algorithm. The frame contains a region of decorrelated data (grey), not untypical when scanning *in vivo*. (a) A single-seed (red) is selected from the *N* alternatives (blue) based on the correlation of the matched windows, as found by brute force search. (b) This seed is used to initialise neighbouring points, whose displacements are then refined. The active point set *S* is shown in red, processed points in green. (c) *S* does not grow into decorrelated region, since the match quality *Q* is low at its boundary, and *S* is processed in order of decreasing *Q*. (d) *S* finds its way around the decorrelated region, unlike row- or column-based approaches, which would need to traverse it. (e) With all the well-correlated data exhausted, *S* will now penetrate the decorrelated region, but the unavoidably poor displacement estimates will not affect the rest of the frame. (For interpretation of the references to colour in this figure legend, the reader is referred to the web version of this article.)

**Fig. 2:**
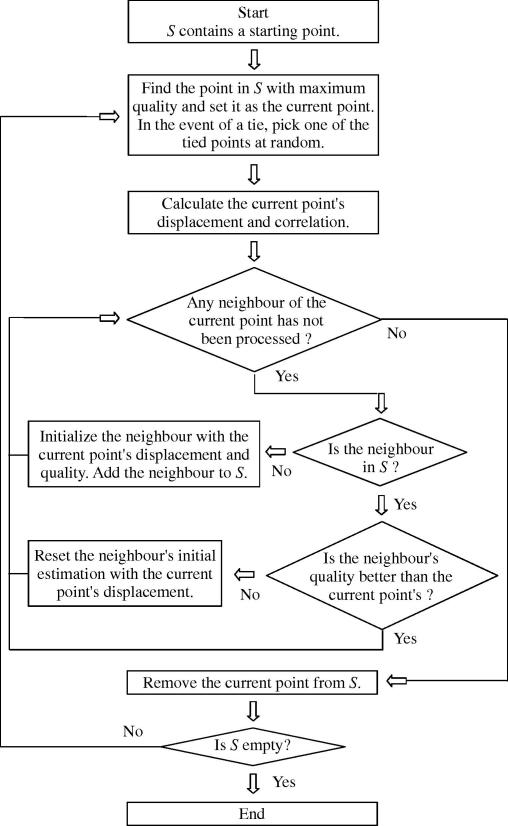
Flowchart of the single-seed quality-guided tracking algorithm.

This strategy ensures that high quality regions are processed first, while low quality regions are avoided at an early stage – see Fig. 1. Accurate displacement estimates in high quality regions are propagated to other points, while the inevitably poor estimates in lower quality regions are prevented from spreading. Note, however, that there is no guarantee that points are processed in order of quality: a high quality point will not be processed until the tracking path reaches it. Nevertheless, since there is no constraint on the direction of propagation, the algorithm is able to process a region with an arbitrary geometrical shape. Fig. 2 shows a flowchart for the single-seed, quality-guided tracking strategy with the correlation quality indicator.

### 2.3 Multiple-seed, quality-guided tracking

There are two common situations that may cause the single-seed tracking algorithm to fail, retrieving only a partially correct displacement map. Firstly, there may be two high quality regions separated by a region of poorly correlated RF data. Whichever region provides the initial seed, it is unlikely that the tracking algorithm will be able to maintain accurate estimation as it traverses the poor quality region. Tracking in the second high quality region will not be correctly initialized and displacement estimation will fail. Secondly, the correct displacement map may be discontinuous, as is often encountered when imaging arterial elasticity (Shi and Varghese, 2007) or, more generally, when there are slip boundaries. When the tracking path reaches a discontinuity where there is a dramatic change in the true displacement, initialization across the boundary is not appropriate and subsequent displacement estimation will fail.

A multiple-seed variant of the quality-guided tracking strategy is proposed to overcome these problems. As with the single-seed version, seed initialization is by the grid point test (Section 2.1), but in this case all the grid points are used as seeds and are added to the point set in the first step:

(6) $$S_{1}=\{p_{1}\text{,}p_{2}\text{,}p_{3}\text{,}\ldots \text{,}p_{N}\}$$

Eq. (6) describes a point set *S* containing *N* seeds. The seeds are not all processed in the first step: instead, only the one with the maximum quality is selected as the current point *Pt*_1_

(7) $${\mathrm{Pt}}_{1}=\arg\max(Q(p_{1})\text{,}Q(p_{2})\text{,}Q(p_{3})\text{,}\ldots \text{,}Q(p_{N}))$$

and fed into the displacement estimation procedure. All subsequent processing – displacement estimation on the current point, initializing the current points’ neighbours, updating the point set, and generating a new current point – is identical to the single-seed version (Fig. 2). If at least one good seed is planted in each disjoint region, all regions should be propagated individually and successfully. This is because the quality at a region’s boundary is relatively low, either because of signal decorrelation or because of discontinuity-induced tracking failure. Hence, when a seed propagates to a region boundary, it stops growing and other seeds get a chance to proceed. Eventually, regions of poorly correlated data, or points at the boundaries between discontinuous regions, are processed at the last stage of tracking. Inevitable estimation errors are confined to these small localities and not propagated elsewhere.

An issue that arises with the use of multiple seeds is the correction of tracking errors caused by bad seeds. Bad seeds arise because the standard cross-correlation metric used for seed initialization can easily pick up an incorrect best match, especially when the window is small or the signal is noisy (Walker and Trahey, 1995): this is known as *peak hopping*. A distinct feature of such seeds is that they are only able to propagate into a small area. The quality at the boundary drops rapidly, and subsequent points are processed by good quality estimates propagating from other seeds. The resulting displacement map, however, contains small regions of incorrect displacement data surrounding the initial poor seeds.

**Fig. 3:**
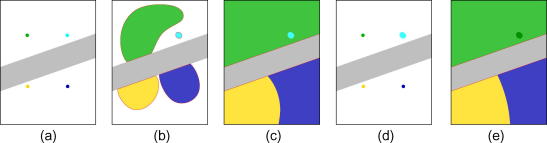
Illustration of the multiple-seed, quality-guided tracking algorithm. The frame contains a band of decorrelated data (grey), that separates the good quality data into two disjoint regions. (a) Only four seeds are shown for clarity: in practice, there will typically be more. Suppose that the green, gold and blue seeds are good, while the cyan seed is bad, even though the correlation is high. (b) The bad seed does not propagate very far, since neighbouring windows do not match well at the incorrect displacement. The three good seeds grow as usual, though they do not penetrate the poorly correlated region. The active point set *S* is shown in red. (c) Towards the end of tracking, all the good quality data has been processed: all that remains is for the green, gold and blue fronts to penetrate the decorrelated region. (d) At the start of the second pass, the bad seed is eliminated, since it grew only a small region in the first pass: this region is flagged for reprocessing (cyan). (e) The green, gold and blue seeds propagate a second time, but no new displacement estimates are calculated in most of the frame: instead, the values found in the first pass are retained. The exception is the small, dark green region, where the first pass displacements are updated using values propagated from the green seed. (For interpretation of the references to colour in this figure legend, the reader is referred to the web version of this article.)

The bad-seed defect can be addressed by re-initializing any problematic regions using good quality points at their boundaries, and repeating the tracking procedure – see Fig. 3. Regions in need of this treatment are identified using a threshold on the number of points propagated from any individual seed: small regions, below the threshold, are labelled as requiring reprocessing. Then, the multiple-seed algorithm is re-run, but this time discarding the seeds that grew the small regions. During this second pass of tracking, displacement estimation is performed only on the labelled regions. This simple method avoids considerable redundant reprocessing and obviates the need to explicitly locate the boundary points of the troublesome regions.

The threshold referred to in the previous paragraph needs to be set to as large a value as possible without running the risk of eliminating valid regions grown from good seeds. Developing this line of reasoning, the threshold can be related to the number of seeds *N* that determines the algorithm’s natural scale. For example, with 20 seeds, we are already conceding that the algorithm cannot be relied on to recover the displacement in disjoint regions occupying less than around 5% of the frame, since such regions are unlikely to be seeded. 5% would therefore be a justifiable threshold for seed elimination too. We accept that regions smaller than this will not be correctly processed, either because they do not contain a seed, or because they are subsequently eliminated before the second tracking pass. If finer scale processing is required, *N* can be increased and the seed elimination threshold reduced by the corresponding amount.

### 2.4 Implementation details

Efficient implementation of the quality-guided tracking algorithm requires each point’s attributes (location, membership of *S*, displacement and quality) to be stored in two separate data structures. The first, a simple two-dimensional array indexed by *x* and *y*, is used to quickly identify neighbours. However, this data structure is unsuitable for one important stage of the algorithm, namely identifying the maximum quality point in the set *S*. Linear search of the two-dimensional array has algorithmic complexity O(n) at each tracking step, where *n* is the number of points.

Thus, the active point set *S* is also maintained as a one-dimensional list, sorted according to quality. The tracking algorithm then need only pick the first point from *S* at each step, with no searching required. When the current point’s neighbours are added to the list, they are inserted at the appropriate location according to their quality, thus maintaining the integrity of the sorted list. Binary search, with complexity O(log(n)), is used to find the insertion indices, significantly reducing the computational load. In such an implementation, the tracking overhead is small compared with the cost of the underlying displacement estimation, even with efficient phase zero search estimation techniques.

## 3 Results and discussion

Field II (Jensen, 1996) simulations and an *in vivo* scan were used to evaluate the quality-guided tracking algorithm. The simulations comprised two RF frames (256 lines, 3464 samples, centre frequency 6.5 MHz, sampling rate 66.67 MHz) with a uniform 1% strain field. Artificially introduced white noise corrupted certain regions of the RF data. Displacement was estimated using windows 10 RF cycles long by five lines wide, spaced at intervals of three RF cycles axially and two lines laterally. The *in vivo* scan of a human carotid artery was recorded using a Terason^3^ T3000 ultrasound system with a 6.25 MHz linear array transducer. The RF sampling frequency was 35.776 MHz, with each frame comprising 128 lines of 2395 samples. Displacement was estimated using windows 10 RF cycles long by three lines wide, spaced at intervals of three RF cycles axially and one line laterally.

In all the studies, we used complex cross-correlation-based exhaustive search, within the maximum expected displacement range, for seed initialization at 20 evenly spaced locations; correlation coefficient as the quality indicator; and phase zero search (Pesavento et al., 1999), with logarithmic compressed signal amplitude, for axial displacement estimation. Lateral displacement was also estimated, by sub-line correlation maximization, within a search range of ±2 lines. Five algorithms were tested, namely exhaustive search without tracking, tracking along A-lines (Pesavento et al., 1999), drop-out correction (Lindop et al., 2008b; Treece et al., 2006, 2008) with a lateral search range of nine windows, and both variants of the quality-guided tracking algorithm.

### 3.1 Tracking of geometrically irregular regions

**Fig. 4:**
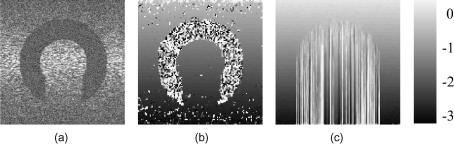
(a) B-mode image of the simulated data. The arc-shaped region contains only noise. Outside the arc-shaped region, white noise is superimposed on the RF signal, with less noise at the centre than at the axial extremities. Displacement obtained by (b) exhaustive search and (c) tracking along A-lines. The units displayed on the right are RF cycles.

Fig. 4a shows a B-mode image of the first simulated data set. The signal in the arc-shaped region is completely masked by white noise, providing a test of the algorithms’ ability to track across decorrelated regions. Noise is also introduced outside the arc-shaped region, with a higher signal-to-noise ratio (SNR) at the centre than at the top and the bottom.

The displacement distribution obtained by exhaustive search is shown in Fig. 4b. Clearly, it is not possible to recover the displacement in the totally decorrelated arc-shaped region. Where the SNR is low (top and bottom of the frame), exhaustive search picks up a large number of false matches and produces erroneous displacement estimates. Results obtained by tracking top-to-bottom along A-lines, starting with an assumed displacement of zero at the top, are shown in Fig. 4c. When the tracking algorithm encounters the uncorrelated data, the displacement estimates inevitably become inaccurate. Since these incorrect estimates are used to initialise the phase zero search at subsequent points, correct displacement tracking is not re-established, even outside the arc-shaped region. The outcome is that tracking errors are propagated from the arc-shaped region downwards. However, it is interesting to note that, except for these understandable tracking errors, the simple A-line tracking strategy performs better than exhaustive search in low SNR regions at the top and bottom of the frame. This illustrates the advantage of exploiting displacement continuity inherent to all tracking approaches.

**Fig. 5:**
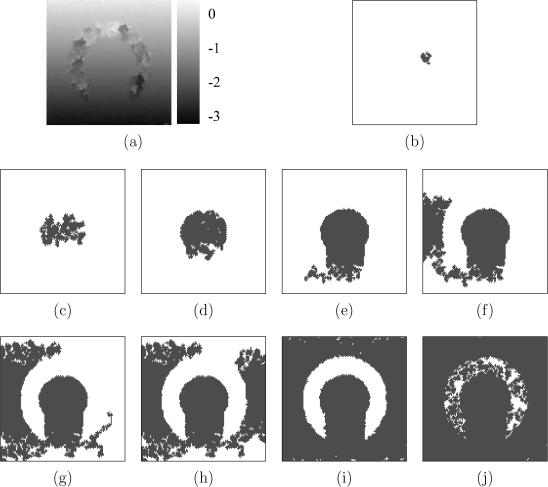
Same data as in Fig. 4a. (a) Displacement obtained by the single-seed quality-guided tracking algorithm. (b) Early displacement propagation from the single-seed. Dark pixels indicate points that have had their displacement estimated by phase zero search, while bright pixels indicate unprocessed points. (c)–(e) Further displacement propagation into the high quality region enclosed by the arc. (f)–(h) Subsequent propagation into remaining high quality regions, reached around the bottom of the arc. (i) When all high quality regions are exhausted, displacement estimates are propagated into the lower quality regions at the top and bottom of the frame, and finally (j) into the noisy arc itself.

The single-seed, quality-guided tracking algorithm retrieves a plausible deformation distribution without any gross errors outside the arc-shaped region – see Fig. 5a. The propagation sequence in Fig. 5b–j exhibits the behaviour anticipated in Fig. 1, with high quality data in the central band processed first, then the lower quality data at the top and bottom, and finally the noisy arc itself. Here, estimation errors are inevitable, but they are prevented from propagating into other regions of the frame. Without constraints on the propagation direction, the quality-guided tracking algorithm is capable of retrieving the displacement distribution of a geometrically irregular region in one pass, while minimizing the effects of estimation errors.

**Fig. 6:**
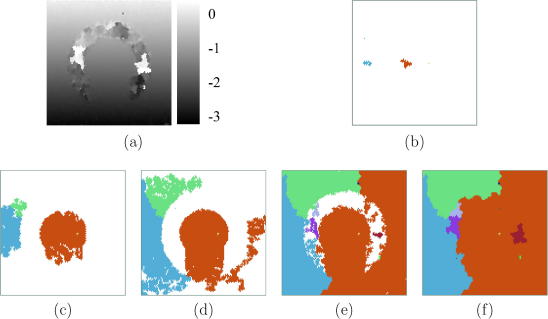
Same data as in Fig. 4a. (a) Displacement obtained by the first pass of the multiple-seed algorithm. (b) Early displacement propagation from two seeds (orange and blue), which were placed in regions of more highly correlated data than the other 18 seeds. (c)–(d) All high quality regions are subsequently propagated from the orange and blue seeds, and a third (green) seed, which grew from a fairly well-correlated region towards the left. (e)–(f) When all high quality data is exhausted, invalid seeds within the arc start to grow. (For interpretation of the references to colour in this figure legend, the reader is referred to the web version of this article.)

While the single-seed variant has performed flawlessly in this case, it is nevertheless instructive to examine the multiple-seed variant as well. In particular, we will see in practice why this algorithm requires two passes to counter the effects of peak hopping errors at the seed initialization stage. Fig. 6a shows the displacement distribution obtained by the first pass. The distribution is mostly correct and errors inside the arc are not propagated. However, a very small patch above the arc has not been correctly processed. The final propagation map (Fig. 6f) shows how this corresponds to a bad seed, whose growth is first evident in Fig. 6e. The bad seed can be traced back to a false correlation peak chosen by the brute force search. Since the correlation peak was nevertheless rather high, the seed had a chance to grow a little before fizzling out as the quality at its boundaries dropped.

**Fig. 7:**
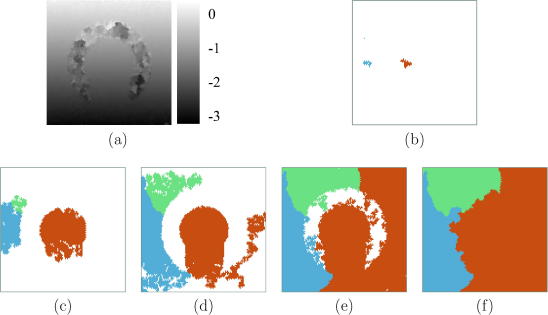
Same data as in Fig. 4a. (a) Displacement obtained by the second pass of the multiple-seed algorithm. (b)–(c) All but three of the original 20 seeds are eliminated since they failed to grow sizable regions in the first pass. (d)–(f) These three seeds propagate across the entire frame, but the displacement is only recalculated in the small regions grown in the first pass from the 17 eliminated seeds. (For interpretation to colours in this figure, the reader is referred to the web version of this paper.)

The second pass of the multiple-seed algorithm fixes the problem with minimal extra computation. A correct displacement distribution is retrieved, as shown in Fig. 7a. The second pass repeats the displacement estimation process on points grown from seeds that form an area smaller than a threshold, in this case 5% of the whole frame.^4^ Initial displacement estimates for these reprocessed regions are obtained at their boundaries using information propagated from other seeds, as illustrated in Fig. 3e. For this particular data set, the 5% test eliminates all but three of the original 20 seeds. Fig. 7b–f show the progress of the second pass: note how the surviving three seeds propagate regions previously processed by the discarded seeds. While Fig. 7b–f hint at similar computational expense to the first pass, this is not in fact the case. The tracking is indeed repeated in its entirety, albeit from a reduced seed set, but the expensive displacement calculations are performed only in the small regions flagged for reprocessing. As explained in Section 2.4, time spent on tracking can be safely ignored in an efficient implementation. The second pass, therefore, is responsible for only a small part of the overall computational cost, as shown in Table 1.

### 3.2 Tracking of disjoint regions

**Fig. 8:**
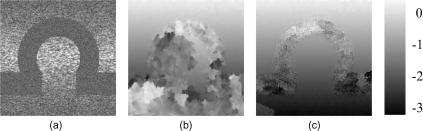
(a) B-mode image of the simulated data. The Ω-shaped region contains only noise. Outside the Ω-shaped region, white noise is superimposed on the RF signal, with less noise at the centre than at the axial extremities. Displacement obtained by (b) the single-seed algorithm and (c) the drop-out correction method.

In the second Field II simulation (Fig. 8a), the noisy arc was extended sideways to partition the well-correlated data into two disjoint regions. Such a situation is not uncommon when scanning *in vivo*, as we shall see in Section 3.3. To succeed, a tracking algorithm will need to recover the displacements in the two regions separately. The single-seed, quality-guided algorithm finds the correct displacement distribution above the arc (where the seed was planted) but is unable to propagate good displacement estimates across the arc and into the lower region – see Fig. 8b. The drop-out correction method chances upon a good solution in this case (Fig. 8c), since one of the spurious displacement estimates at the bottom edge of the arc just happens to be within a wavelength of the correct value. This good estimate is then propagated downwards and sideways in the first pass, and most of the remaining poor estimates are corrected in the second pass. However, erroneous displacement estimates remain at the bottom left corner of the frame.

**Fig. 9:**
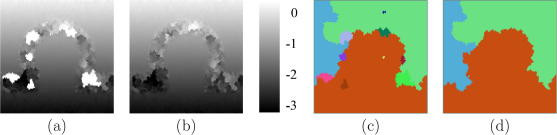
Same data as in Fig. 8a. Displacement obtained by the first (a) and second (b) passes of the multiple-seed algorithm. Seed propagation maps from the (c) first and (d) second passes. (For interpretation to colours in this figure, the reader is referred to the web version of this paper.)

A multiple-seed approach is necessary for reliable tracking in such situations. Fig. 9a shows the displacement distribution retrieved by the first pass of the multiple-seed tracking algorithm. Accurate displacement estimates are obtained throughout the two well-correlated regions. The seed propagation map in Fig. 9c shows how spurious seeds inside the noisy region get a chance to grow towards the end of the tracking process, producing the bright patches in Fig. 9a.

These white patches fall below the 5% threshold and are therefore among the regions reprocessed in the second pass of the multiple-seed algorithm (Fig. 9b), which starts from just three seeds (Fig. 9d). Although in this case reprocessing is not absolutely necessary, in general the second pass does help to correct discontinuous regions grown from bad seeds. Comparing the propagation maps of the two passes, it is apparent that the three seeds used in the second pass have invaded some of each other’s territory, as well as that of the eliminated seeds. However, the displacement is only recalculated in those small regions flagged for reprocessing. Doing otherwise – for example, overwriting good quality displacements on a per-point basis with better quality displacements – would not only slow down the algorithm but also introduce unwanted, small discontinuities in otherwise continuously tracked regions.

### 3.3 *In vivo* displacement tracking

**Fig. 10:**
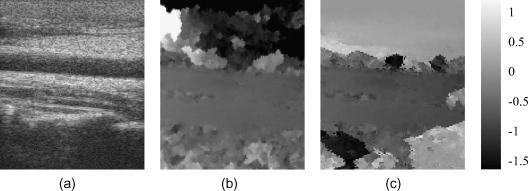
(a) B-mode image of the carotid artery scan. Displacement obtained by (b) the single-seed algorithm and (c) the drop-out correction method.

Fig. 10a shows a B-mode image of a human carotid artery. The artery separates well-correlated data into two regions. Inside the artery, the scattering is weak and blood flow causes significant decorrelation between the pre- and post-deformation frames. A further challenge to displacement estimation is the pulsation of the arterial wall, which produces a discontinuous deformation distribution in its vicinity. Both these factors point to the need for a robust tracking algorithm. Neither the single-seed algorithm (Fig. 10b) nor the drop-out correction method (Fig. 10c) succeed in this case. While one would not expect good displacement estimation within the artery or in the noisy region at the bottom of the frame, the single-seed algorithm has failed to recover the displacement above the artery (since the seed was below it), while the drop-out correction method clearly fails in a small region at the right of the frame, just below half way down.

**Fig. 11:**
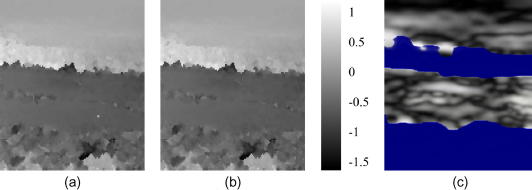
Same data as in Fig. 10. (a) Displacement obtained by the first (a) and second (b) passes of the multiple-seed algorithm. (c) Strain image derived from (b), with black and white representing 0% and 0.5% strain respectively. The blue mask suppresses display of strain in low quality regions. (For interpretation of the references to colour in this figure legend, the reader is referred to the web version of this article.)

The first pass of the multiple-seed tracking algorithm produces promising results, with plausible displacements both above and below the artery – see Fig. 11a. However, there is a visible error – a tiny bright dot – below the artery in Fig. 11a, just to the right of centre. This is the result of a bad seed, which managed to grow a little in the later tracking stages when most of the high quality data had been exhausted. Fig. 11b shows how this error is corrected, at small computational expense (Table 1), by the second pass. Fig. 11c shows the resulting strain image, with a blue colour wash obscuring regions where the correlation falls below a user-defined threshold.

### 3.4 The effect of SNR and window length

It remains to compare the various tracking strategies in terms of their robustness to different SNR levels and different RF window lengths. For any given amount of strain and SNR, there are lower and upper bounds on the RF window lengths that allow accurate displacement estimation (Lindop et al., 2008b). Too short a window and the correct match will have much the same correlation as incorrect matches one or more RF cycles away: this is especially evident at low SNR. Conversely, too long a window and intra-window deformation will lower the correlation of the correct match, making it less distinguishable from incorrect matches: this is especially evident at high strain.^5^ We demonstrate in this section that the tracking algorithm plays its part too: more sophisticated approaches allow a wider range of window lengths for any given strain and SNR.

Two RF frames were simulated with uniform echo amplitude and a 1% strain field. Random white noise was added at five SNR levels (4 dB, 2 dB, 0 dB, −2 dB and −4 dB) to evaluate the tracking capability. Unlike the previous tests, there is no completely decorrelated region and the SNR is uniform across each frame. The experiment can therefore focus on the core ability of each tracking algorithm to recover a continuous displacement field in the presence of noise, without the extra challenges of severe, local data decorrelation and displacement discontinuity.

**Fig. 12:**
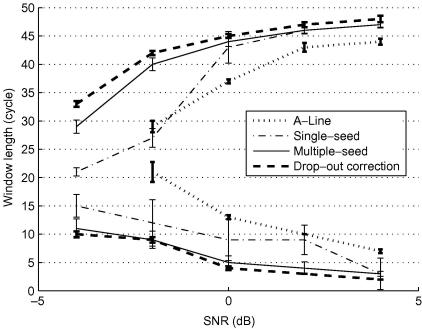
Effective tracking range. The plot shows upper and lower bounds on window lengths for effective tracking at a number of noise levels. The ±1 standard deviation error bars are based on 20 repetitions.

At each SNR level, four tracking strategies were tested: tracking along A-lines, the drop-out correction method, the single-seed, quality-guided algorithm and the multiple-seed, quality-guided algorithm. The RF window length of the underlying displacement estimation algorithm (phase zero search) was varied between 1 and 50 cycles. We deemed each experiment a success if more than 90% of the displacement estimates were correct, where ‘correct’ means within a quarter cycle of the known, true value. Although arbitrary, this criterion allows a meaningful comparison of the various tracking algorithms. As expected, at each noise level, there were clear upper and lower bounds on the window lengths that allowed successful recovery of the displacement field. These bounds are plotted in Fig. 12. The area enclosed by the lower and upper bounds provides a measure of each algorithm’s robustness to different SNRs and window lengths. We call this area the *effective tracking range*. Although the results in Fig. 12 are based on 1% strain, other strain levels produced similar patterns, as did other success thresholds (we tried 75% and 95%).

The simplest strategy, tracking along A-lines, has the smallest effective tracking range. If an estimation error occurs, the method has no mechanism to prevent it from propagating. The single-seed algorithm performs poorly as well. At low SNR levels (below 0 dB), the single seed is often bad, even though it has the highest quality amongst all the grid points. When this happens, tracking fails immediately and never recovers. This is an obvious brittleness of the single seed approach that we have deferred mentioning until now.

Although the single-seed algorithm is superior to the drop-out correction method in tracking a geometrically irregular region, its weakness lies in its poor tolerance of noise. The latter, on the other hand, is more robust against noise because it searches several previously processed points for the best initial guess at each new point. The fragility of the single-seed algorithm is overcome in its multiple-seed variant. If the region grown from one seed goes wrong, others can still proceed correctly. The multiple-seed algorithm has a comparable effective tracking range to that of the drop-out correction method, with the advantage that it can process disjoint and irregularly shaped regions.

## 4 Conclusion

Displacement tracking is an algorithmic procedure implicit in the data processing pipelines of all ultrasonic strain imaging systems. Despite playing a large part in the efficiency and stability of the displacement estimation process, previous work on tracking has been tightly interwoven with particular displacement estimation techniques. In this paper, a generic, quality-guided tracking framework has been proposed. It can be integrated with a wide variety of displacement estimation techniques, including correlation maximization and phase zero search. The single-seed algorithm demonstrates the fundamental working mechanism of the quality-guided tracking strategy. The multiple-seed variant greatly enhances the algorithm’s robustness to noise and data discontinuity.

Further research on displacement tracking should explore alternative quality indicators. The measure used in this study, the complex cross-correlation coefficient, was generally effective but less so at low SNR. Alternative approaches, perhaps including a continuity element, might perform better in high noise situations. Another area for further research concerns the size of the RF windows. Sophisticated tracking strategies allow the use of shorter RF windows, and some classes of post-filtering methods (for strain estimation and noise rejection) promise to work particularly well with short windows (Lindop et al., 2008a). Finally, the multiple-seed version of the quality-guided tracking algorithm produces, as a by-product, an approximate segmentation of the frame into regions of continuous displacement. This segmentation could be refined and then used to improve the subsequent calculation of strain. Specifically, differentiation across displacement discontinuities could be avoided, thus eliminating some of the artefacts that commonly affect strain images.

## Figures and Tables

**Table 1:** Execution time of the multiple-seed displacement tracking algorithm, running single-threaded on a 2.4 GHz Intel Core 6600 processor. The simulations were slower since they employed twice as many RF lines and wider windows for displacement estimation. The timings for each pass are divided into tracking (choosing the next point to be processed and maintaining the data structures described in Section 2.4) and displacement estimation (in this case by phase zero search).

Component	Time (ms)	
	Simulation	*In vivo*
Preprocessing (compute baseband analytic signal)	46	16
Seed initialization	0.5	0.5
First pass tracking	5	5
First pass displacement estimation	46	23
Second pass tracking	5	5
Second pass displacement estimation	2	2

## Footnotes

1 An alternative approach is direct strain estimation (Alam et al., 1998).

2 Hereafter, we shall refer loosely to each RF window as a *point*. Displacement is estimated at these points, then differentiated to obtain strain. A displacement tracking algorithm propagates displacement estimates from point to point.

3 www.terason.com.

4 This is not an arbitrary threshold, but tied to the number of seeds (20, in this case) that determines the algorithm’s natural scale, as explained in Section 2.3.

5 While this effect can be ameliorated by adaptive stretching (Céspedes et al., 1993), this comes at a high computational cost. Moreover, there is strong evidence to suggest that such an approach is not necessary for high accuracy displacement estimation (Lindop et al., 2007).
